# Near vision assessment for adults using the NIH Toolbox

**DOI:** 10.3389/fneur.2025.1533382

**Published:** 2025-01-20

**Authors:** John-Christopher A. Finley, Jerry Slotkin, Cindy J. Nowinski, Katy Bedjeti, Nicholas Volpe, Sandra Weintraub, Richard Gershon

**Affiliations:** ^1^Department of Psychiatry and Behavioral Sciences, Feinberg School of Medicine, Northwestern University, Chicago, IL, United States; ^2^Center for Health Assessment Research and Translation, University of Delaware, Newark, DE, United States; ^3^Department of Medical Social Sciences, Feinberg School of Medicine, Northwestern University, Chicago, IL, United States; ^4^Department of Neurology, Feinberg School of Medicine, Northwestern University, Chicago, IL, United States; ^5^Department of Ophthalmology, Feinberg School of Medicine, Northwestern University, Chicago, IL, United States; ^6^Mesulam Center for Cognitive Neurology and Alzheimer’s Disease, Feinberg School of Medicine, Northwestern University, Chicago, IL, United States

**Keywords:** near vision, visual acuity, NIH Toolbox, cognitive, neurology, neuropsychology

## Abstract

**Introduction:**

The National Institutes of Health (NIH) Toolbox Near Visual Acuity Test is a novel digitized measure designed to provide an assessment of near vision in a time-and cost-effective manner. This study is the first to report the psychometric properties of the NIH Toolbox Near Visual Acuity Test in a sample of community-dwelling middle-aged and older adults.

**Methods:**

Ninety-eight adults (ages 40–81) completed the tablet-based near vision test and the gold standard chart-based near vision test. Performance on the tablet-and chart-based near vision tests was expressed in logarithmic units. Chart-and tablet-based administration order was counterbalanced. To assess test–retest reliability, the NIH Toolbox Near Visual Acuity Test was administered twice within the same day. Additionally, two equivalent versions of the chart-based test were randomly assigned to participants.

**Results:**

Analyses revealed that test–retest reliability of the NIH Toolbox Near Visual Acuity Test was good (intraclass correlation = 0.87, *p* < 0.001). Concurrence between the NIH Toolbox Near Visual Acuity Test and gold standard chart-based test was also good (*r* = 0.79, *p* < 0.001).

**Discussion:**

Findings provide support for the reliability and validity of the NIH Toolbox Near Visual Acuity Test as a near vision assessment for middle-aged to older adult populations. With further research, the integration of this test within the widely used NIH Toolbox may provide a more efficient means to understanding how near visual acuity influences neurocognitive test performance and brain function in middle-aged to older adult populations.

## Introduction

Near Vision (NV), the ability to see details up close, is necessary for many instrumental daily activities such as reading medication labels and financial bills (1). NV may also be informative of changes in brain function (2, 3). Research suggests that reduced NV in middle-aged and older adults is predictive of cognitive decline, dementia, and neurodegenerative disease (4–13). Furthermore, reduced NV may interfere with performance on visually based neurocognitive tests that are used to make judgments about brain function (14–16). For these reasons, it has become increasingly recognized that NV should be examined in studies focused on brain health in middle-aged and older adults; despite this, assessment of NV in research remains an uncommon practice (2).

One reason NV, as assessed by near visual acuity, may not be regularly examined in research is that standard chart-based assessments may be somewhat burdensome for scientists who are considering ways to assess multiple brain health variables. Chart-based tests must be administered by trained and certified examiners who manually record and convert the responses into specific notations to quantify visual acuity. Such scores must then be manually entered into a database. Collectively, these factors may limit the use of chart-based vision assessment methods in research, particularly when vision is not the primary study focus. These barriers have led some researchers to rely on self-reported visual ability (17); but assessing NV via self-report has shown to be less accurate than chart-based measures, especially in cognitively impaired populations where NV assessment may be much needed (18–20).

Alternatively, researchers have attempted to mitigate the demands of NV assessment by using digitized measures (21–23), which have the potential to reduce administration time and recording errors. The automated aspect of these measures allows them to potentially maintain a high degree of reliability and accuracy. Furthermore, digitized measures can include software that automatically transfers data to repositories at the point of testing, allowing for more streamlined data collection and maintenance (24). Yet, very few digitized measures have been psychometrically validated in adult populations (22, 23), and none has been co-developed with a battery of neurocognitive tests used to assess brain function. Creating a NV measure within a neurocognitive test battery allows for concurrent norming and validation across all measures within the same sample. Such an approach would ensure consistent testing conditions, minimize variability from sample characteristics, and yield directly comparable scores across NV and other brain health measures. With the emergence of digitized neurocognitive testing (24, 25), developing a valid and reliable digitized NV test for concurrent use may become increasingly relevant.

The developers of the National Institutes of Health Toolbox for Assessment of Neurological and Behavioral Function (26) (NIH Toolbox®, or NIHTB) have recently developed the Near Visual Acuity Test (NVAT), an iPad-based measure designed to provide an automated, cost-effective assessment of NV that does not require trained ophthalmic technicians or optometrists. The test is integrated within the NIHTB, a widely used research test battery for assessing cognitive abilities as well as motor, emotional, and sensory functioning, including distance vision (26, 27). This new test has the potential not only to mitigate some issues that have limited the assessment of NV in research, but also to enable a more direct evaluation of how such vision affects performance on other visually based and digitized tests of brain function. The NIHTB NVAT is not designed to replace chart-based measures, but instead to offer an accessible means of NV assessment, especially for researchers who already use the NIHTB or who are interested in studying or measuring vision concurrently with cognition and other sensory abilities. With these advancements, researchers may be better positioned to assess and predict changes in brain health among middle-age to older adults. Prior to the work detailed herein, the NIHTB NVAT had not yet been validated in any population, including middle-aged and older adults who are most likely to experience age-related visual and cognitive decline (28). Thus, the current study reports an initial validation of the NIHTB NVAT in a sample of community-dwelling middle-aged and older adults. We have used a cross-sectional design to examine the test–retest reliability and concurrent validity of the NIHTB NVAT, as compared to the gold standard chart-based NV test.

## Materials and methods

### Participants

One hundred and three community-dwelling participants were recruited in a large Midwestern city via a market research firm with extensive experience recruiting for similar studies. Inclusion criteria were adults living in the community ages 40 or older. Exclusion criteria were (1) presence of visual impairment due to disease or injury that could not be corrected to normal in both eyes with glasses/contact lenses; (2) history of neurologic condition including neurodegenerative disease; (3) cognitive impairment as evidenced by an inability to understand informed consent, or history of diagnosis of dementia. Exclusion and inclusion criteria were applied based on participant self-report.

After applying these criteria, the final sample comprised 98 participants. The sample was equally divided between sex (52% male). Average age was 62.69 years (range 40–81). Most participants (70%) identified as non-Hispanic White and had a middle-to-upper class household income (56% of the sample reported annual household income of $75,000 or higher). 98% of the sample rated their overall health as “good” to “excellent.” A minority of the sample (12%) reported having an eye condition that was present at the time of the study, with astigmatism being the most commonly reported. 27% of the sample reported a history of eye surgery, with some individuals having undergone multiple surgeries (refer to Supplementary Table 1 for the count of specific types of surgeries). Details regarding other sample demographics and characteristics are provided in Table 1 and Supplementary Table 1. Data for this IRB-approved study were collected in 2023.

**Table 1:** Sample characteristics and descriptive statistics.

Demographics and characteristics	Means and proportions
Age	*M* = 62.69 (SD = 11.96; range = 40–81)
Male sex	51 (52%)
Racial / Ethnic identity	
Non-Hispanic White	69 (70%)
Hispanic	13 (13%)
Non-Hispanic Black	11 (12%)
Asian	3 (3%)
Other	2 (2%)
Employment status	
Working full time	42 (43%)
Working part time	15 (15%)
Retired	34 (35%)
Unemployed	6 (6%)
Missing	1 (1%)
Household income	
$10,000–$19,999	4 (4%)
$20,000–$39,999	9 (9%)
$40,000–$74,999	25 (26%)
$75,000–$99,999	16 (16%)
≥ $100,000	39 (40%)
Missing	5 (5%)
General health	
Excellent	21 (21%)
Very good	43 (44%)
Good	32 (33%)
Fair	1 (1%)
Poor	0 (0%)
Missing	1 (1%)
Participants with current eye condition(s)	12 (12%)
Participants with history of eye operation(s)	26 (27%)

M, mean; SD, standard deviation.

### Procedure

Before beginning the NV assessment, participants provided written informed consent and completed a self-report questionnaire regarding medical history and sociodemographic information. To simulate everyday reading, participants were also asked to wear any corrective lenses (contact lenses, prescription or over-the-counter reading glasses) that they would typically wear for reading and all testing was done binocularly. For the NV assessment, participants were then seated at a table in an enclosed, quiet room with the iPad screen or paper chart positioned 16 inches (40 cm) from them. Both the chart and iPad were placed on a stand with an attached string to ensure the correct distance from the participant to the test stimuli. To replicate a naturalistic environment, participants were asked their preferred reading angle from the table, to match how they read in other settings. They could choose either a 0-, 30-, 45-, 60-, or 90-degree angle. Participants were instructed to maintain their preferred angle and distance without moving their head or body forward or backward during the assessment.

Participants were administered two types of visual acuity tests: the iPad-based test (NIHTB NVAT) and a paper chart-based test (Logarithmic Visual Acuity Chart 2000). Certified ophthalmic technicians administered all the tests to ensure accurate administration of the chart-based test. Although technicians were not needed for the NIHTB NVAT, they were present to avoid switching examiners during the assessment process. Chart-and iPad-based test administration order was counterbalanced. After the initial administration of both vision tests, the iPad-based test was administered another time to evaluate test–retest reliability. In summary, the iPad-based test was administered twice and paper chart-based test was administered once. Both the iPad-and chart-based tests had two equivalent forms with varying letter arrangements, randomly alternating between participants. Participants were given a 3-minute break between administration of each of the 3 test conditions to minimize testing fatigue. Similar instructions were provided for each visual acuity test, asking participants to identify letters on the screen or chart and guess the letter if they were uncertain. Participants were told the presented letters would decrease in size so that the examiner could determine their smallest readable size.

### Measures

#### Paper chart-based near vision test

The Logarithmic Visual Acuity Chart 2000 was used as the paper chart-based near vision test. This chart is consistent with the Early Treatment of Diabetic Retinopathy Study design (29) and is considered the gold standard test for near visual acuity in clinical research (30). This paper chart-based test consists of 16 lines with five optotypes (letters) per line that descend from biggest to smallest in size. Administration begins with a screening phase to approximate the participant’s visual acuity threshold, and then proceeds with a testing phase to index their actual near visual acuity. During the screening phase, participants are instructed to identify the first letter presented on each line. They begin with the logarithm of the minimum angle of resolution (logMAR) size of 0.7 (or 20/100 in Snellen notation) and continue either smaller until they can no longer correctly identify the first letter of each line, or larger until they can identify the first letter on the line. During the testing phase, participants are presented with five letters per line, starting at the line of the smallest readable letter identified in the screening. If participants accurately identify at least 3 of 5 letters on a line, they are asked to read lines with smaller, more challenging letters until they cannot identify at least 3 of 5 on a line. If they identify fewer than 3 of 5 letters correctly, they are presented with larger, easier lines until they accurately identify at least 3 of 5 letters. The objective is to identify the line with the smallest letters where the participant accurately reads at least 3 of 5 letters.

Visual acuity performance can be calculated in two ways. The first is the logMAR notation. This notation uses the logarithm of the angular size of the smallest discernible letter that examinees can identify. It is based on a proportional scale where each step represents a tenfold increase or decrease in acuity. The logMAR value for each line on a chart is calculated by taking the logarithm (to base 10) of the angular size in minutes of arc of that line’s letters at a standard distance. This scale inversely relates to visual acuity. Lower logMAR scores indicate better visual acuity, while higher scores indicate poorer acuity. LogMAR scores for near visual acuity can range from −0.3 to 1.3 and progress in increments of 0.1. For the current study, optotypes corresponding to logMAR values of 0.0 to 1.3 were used, since the iPad version of the assessment could accurately produce letters as small as logMAR = 0.0 due to screen size and resolution factors. The second way of calculating visual acuity performance is via the commonly used Snellen notation. This notation is based on a ratio that corresponds to the line with the smallest letters on which participants accurately identified at least 3 of 5 letters. Snellen for near vision is expressed as a ratio, ranging from 20/20 to 20/400 for this study. A logMAR of 0.0 corresponds to a Snellen of 20/20. A 0.1 increase in logMAR results in a decrease in Snellen acuity, and vice versa. Thus, with each step in logMAR, there is a corresponding, predictable change in the Snellen ratio that indicates a person’s visual acuity. Both logMAR and Snellen were calculated for descriptive purposes since they are commonly used visual acuity notations; however, only logMAR scores were used in the analyses because they are a more precise index of visual acuity (30, 31).

#### iPad-based near visual acuity test

The NIHTB NVAT is programmed to be administered via an iPad. Administration of the iPad-based test is more straightforward than the chart-based test. During the assessment procedure described above, participants are asked to vocally identify each letter as it is displayed one at a time on a screen. Responses are recorded via a wireless keyboard as correct or incorrect by the administrator. There is no time limit, but testing typically takes 2–3 min to complete. The iPad automatically discontinues testing once scoring thresholds have been reached and records participants’ vision scores on the database that is stored within the iPad. Just as with the paper chart-based test, the iPad-based test expresses near visual acuity performance as logMAR and Snellen scores. Scoring of the iPad administered test is automated, reducing potential for error. This test also allows for other features, including recording of the letter presentation sequence and automatically randomized assignment of the two equivalent optotype charts.

### Statistical analyses

*Post-hoc* power analyses indicated an observed power > 80% for all findings. Test–retest reliability was calculated using intraclass correlation coefficients (ICCs) between results from the first and second administration of the NIHTB NVAT. Correlations were also run to identify any potential differences in test–retest reliability based on sequence of administration. Concurrent validity of the iPad-based NV test scores with those obtained from the paper chart-based NV test was evaluated with a Pearson (*r*) correlation coefficient.

## Results

### Testing preferences

As shown in Table 2, test administration order was counterbalanced, with 54% of participants starting with the iPad-based test and 46% with the chart-based test, each followed by alternate versions of the tests. Approximately half of the participants (51%) preferred the 60-degree angle for test placement, while 21% selected the 90-degree angle. The 30-degree and 45-degree angles were less preferred, at 13 and 12% respectively, and flat placement (zero degrees) was selected by only one participant. During the testing, most participants (76%) wore corrective lenses for reading purposes.

**Table 2:** Near vision testing preferences and performance.

Testing preferences and performance	Proportions
Order of test administration	
iPad-chart-iPad	53 (54%)
Chart-iPad-iPad	45 (46%)
Test angle placement	
30-degree	13 (13%)
45-degree	12 (12%)
60-degree	50 (51%)
90-degree	21 (21%)
Flat	1 (1%)
Missing	1 (1%)
Wore corrective lenses	74 (76%)
Near vision performance – LogMAR	
NIH Near Visual Acuity Test (1st Administration)	*M* = 0.07 (SD = 0.09; range = 0.00–0.48)
NIH Near Visual Acuity Test (2nd Administration)	*M* = 0.07 (SD = 0.08; range = 0.00–0.48)
Logarithmic visual acuity chart 2000	*M* = 0.09 (SD = 0.11; range = 0.00–0.60)

M, mean; SD, standard deviation; LogMAR, logarithm of the minimum angle of resolution.

### Near visual acuity scores

The average logMAR visual acuity scores were 0.07 for both the first and second administrations of the NIHTB NVAT and 0.09 for the chart-based test, which approximately correspond to Snellen scores of 20/20 and 20/25, respectively. Because logMAR values of 0.0 and Snellen values of 20/20 are considered ‘normal’ visual acuity, we did not provide a more refined assessment beyond these values.

### Test–retest reliability

**Figure 1:**
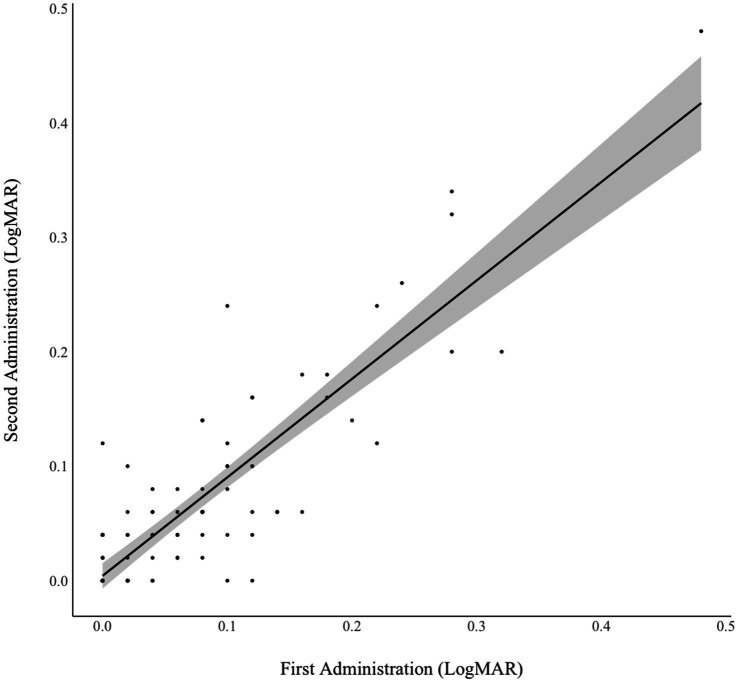
Test–retest reliability of NIH Toolbox Near Visual Acuity Test. Intraclass correlation coefficient = 0.87, *p* < 0.001.

As shown in Figure 1, test–retest reliability of the NIHTB NVAT was good (ICC = 0.87, *p* < 0.001).

### Concurrent validity

**Figure 2:**
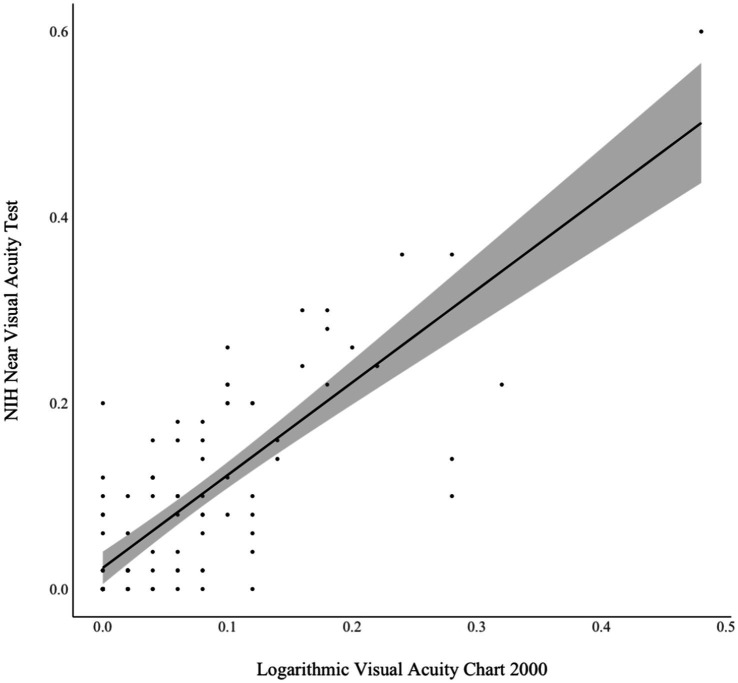
Concurrent validity of NIH Toolbox Near Visual Acuity Test and chart-based near visual acuity test. Pearson *r* = 0.79, *p* < 0.001.

As shown in Figure 2, analyses also revealed good concurrence between the NIHTB NVAT and gold standard chart-based test scores (*r* = 0.79, *p* < 0.001). This association did not vary significantly (*p* > 0.05) between participants who did (*r* = 0.79) and did not (*r* = 0.84) wear corrective lenses.

## Discussion

This study evaluated the reliability and validity of a new measure of NV, known as the NIHTB Near Visual Acuity Test (NVAT). A cross-sectional design was used to assess the test–retest reliability and concurrent validity of the NIHTB NVAT, as compared to the gold standard chart-based NV test, in a community-dwelling sample of middle-aged to older adults. Overall, these initial findings provide support for the use of the NIHTB NVAT as a measure of NV for middle-aged to older adult populations.

The NIHTB NVAT is unique in many ways. To begin, it is among the few digitized NV tests to be validated in an adult population. By employing a tablet-based approach with automated administration and scoring that is completed within 2–3 min, this test circumvents the training and time requirements associated with administering chart-based tests. The NIHTB NVAT may also allow for more equitable assessment in both community-based and clinical research settings since it is low-cost. From a feasibility standpoint, this test offers a valid and easy way to assess NV in adult-focused research, since it is integrated into the widely used NIHTB. This digital measure of NV automates data collection and entry at the point of testing, which eliminates any imputation errors or inconsistencies in the dataset and allows researchers to access and utilize the data in a timely manner. The NIHTB NVAT software can also facilitate multidisciplinary research efforts, as the data can automatically transfer to secure and centralized cloud-based repositories that are accessible to various stakeholders across different settings.

The integration of this test within a digital neurocognitive test battery is also important. By integrating this digitized test within the NIHTB, researchers can elucidate the complex association between NV and cognitive/brain health more efficiently and effectively. Specifically, it enables a more direct examination of how NV influences visually based neurocognitive test performance in adults who commonly experience age-related visual decline. In all applications of neurocognitive testing, for either screening or comprehensive evaluation purposes, scientists and practitioners must consider potential sources of error variance that hinder performance on such testing. Visual deficits may be one source of error variance that warrants further consideration. This may be especially important to consider for researchers using the NIHTB tests to assess neuropsychological status in adults, as most of the NIHTB cognitive tests are visually based. With further research, scientists may begin to develop contrast scores that differentiate between performance on NV and neurocognitive tests, helping practitioners elucidate a potential source of error variance from true disease pathology. By using the same digital interface (i.e., an iPad screen) for the NIHTB NVAT and the cognitive tests, researchers may have the means to better investigate the association between NV and cognitive test performance. Thus, the integration of this NV test within the NIHTB may serve as another tool to help detect changes in brain function or early signs of neurodegenerative disease in middle-aged to older adults.

The strong test–retest reliability and concurrent validity of the NIHTB NVAT suggest that this test possesses the psychometric properties required for NV assessment in research. The reliability and validity values found in this study are highly consistent with those reported in the validation study of the NIHTB distance vision test (27). High reliability has also been reported in the few other existing tablet-based distance vision tests (22). Because the NIHTB Visual Acuity Test (measuring distance vision) (27) and NVAT both have strong psychometric properties and can be administered from the same software application and tablet device, the tests may eventually be used together to provide a brief assessment of near and distance vision. Nonetheless, further replication of these findings in independent samples is needed before the NIHTB NVAT is used clinically.

Indeed, the current study is not without limitations. The study sample was demographically homogeneous, skewing toward white adults with middle-to upper-class household incomes. It would be particularly helpful to include a larger and more diverse sample of adults to more firmly establish test sensitivity and specificity when testing patients with cognitive impairment as well as reduced visual acuity. Future researchers might also consider using standardized measures to define the inclusion and exclusion criteria for study participants. Participants in the current study may have underreported certain inclusion and exclusion criteria, such as those related to a history of dementia or eye disease, given that such criteria were determined via unstandardized and self-reported measures. Formal screening measures could have also been used to more accurately determine which participants had simple or easily identifiable eye-related issues (e.g., ocular motor issues) that are known to affect NV; however, eye-related issues were not of much concern in the current study given that participants were asked to wear corrective lenses and most participants performed within normal limits on the NV testing. Moreover, given the cross-sectional nature of this study, the predictive validity of the test for longitudinal changes in NV and cognitive function remains to be established. Finally, the NIHTB NVAT would be inappropriate for individuals who are unfamiliar with the English alphabet or are unable to accurately name the letters on the screen. It is also possible that performance on the NIHTB NVAT may vary according to socioeconomic status and other social determinants of health; however, the size of the study sample precluded our ability to analyze these potential confounding factors.

The psychometric characteristics reported in this study suggest that the NIHTB NVAT is a valid and easy way to assess NV in middle-aged and older adults. An important facet of this test comes from the understanding that sensory and cognitive abilities are interrelated (32), and integrating tests of these abilities is crucial for evaluating brain health. As such, the integration of this NV test within the widely used NIHTB may provide another means to understanding how visual acuity influences neurocognitive test performance as a measure of brain health.

## Data Availability

The raw data supporting the conclusions of this article will be made available by the authors, without undue reservation.
