# Supplementary material for: Near vision assessment for adults using the NIH Toolbox
Source: Front Neurol. 2025 Jan 20;16:1533382. doi: 10.3389/fneur.2025.1533382 (PMC11788145; doi:10.3389/fneur.2025.1533382)
Supplement: Supplementary file 1 [file Data_Sheet_1.pdf]

Supplemental Table 1 Additional sample characteristics.

Characteristics	Proportions
Type of current eye conditions	
Cataracts	2 (2%)
Astigmatism	4 (4%)
Dry eyes	3 (3%)
Color blind	1 (1%)
Floaters	1 (1%)
Macular degeneration	1 (1%)
Type of historical eye operations [a]	
Cataract surgery	16 (16%)
Lasik	9 (9%)
Vitrectomy	2 (2%)
Strabismus surgery	1 (1%)
Reconstructive surgery for eyelid cancer	1 (1%)
Lens replacement	1 (1%)
Most recent eye operation	
≤ 6 months	1 (1%)
≤ 12 months	1 (1%)
> 12 months	24 (24%)

[a] These values represent the surgical procedures in Table 1 that were reported by the 26 participants, some of whom had undergone multiple procedures.
